# Echo Chamber Effect in Rumor Rebuttal Discussions About COVID-19 in China: Social Media Content and Network Analysis Study

**DOI:** 10.2196/27009

**Published:** 2021-03-25

**Authors:** Dandan Wang, Yuxing Qian

**Affiliations:** 1 School of Information Management Wuhan University Wuhan China; 2 Center for Studies of Information Resources Wuhan University Wuhan China; 3 Big Data Institute Wuhan University Wuhan China

**Keywords:** rumor rebuttal, infodemiology, infodemic, infoveillance, echo chamber effect, attitude, COVID-19, Weibo

## Abstract

**Background:**

The dissemination of rumor rebuttal content on social media is vital for rumor control and disease containment during public health crises. Previous research on the effectiveness of rumor rebuttal, to a certain extent, ignored or simplified the structure of dissemination networks and users’ cognition as well as decision-making and interaction behaviors.

**Objective:**

This study aimed to roughly evaluate the effectiveness of rumor rebuttal; dig deeply into the attitude-based echo chamber effect on users’ responses to rumor rebuttal under multiple topics on Weibo, a Chinese social media platform, in the early stage of the COVID-19 epidemic; and evaluate the echo chamber’s impact on the information characteristics of user interaction content.

**Methods:**

We used Sina Weibo’s application programming interface to crawl rumor rebuttal content related to COVID-19 from 10 AM on January 23, 2020, to midnight on April 8, 2020. Using content analysis, sentiment analysis, social network analysis, and statistical analysis, we first analyzed whether and to what extent there was an echo chamber effect on the shaping of individuals’ attitudes when retweeting or commenting on others’ tweets. Then, we tested the heterogeneity of attitude distribution within communities and the homophily of interactions between communities. Based on the results at user and community levels, we made comprehensive judgments. Finally, we examined users’ interaction content from three dimensions—sentiment expression, information seeking and sharing, and civility—to test the impact of the echo chamber effect.

**Results:**

Our results indicated that the retweeting mechanism played an essential role in promoting polarization, and the commenting mechanism played a role in consensus building. Our results showed that there might not be a significant echo chamber effect on community interactions and verified that, compared to like-minded interactions, cross-cutting interactions contained significantly more negative sentiment, information seeking and sharing, and incivility. We found that online users’ information-seeking behavior was accompanied by incivility, and information-sharing behavior was accompanied by more negative sentiment, which was often accompanied by incivility.

**Conclusions:**

Our findings revealed the existence and degree of an echo chamber effect from multiple dimensions, such as topic, interaction mechanism, and interaction level, and its impact on interaction content. Based on these findings, we provide several suggestions for preventing or alleviating group polarization to achieve better rumor rebuttal.

## Introduction

### Background

In the early stage of the COVID-19 crisis, social science played an essential role in the containment of the disease [1]. The core part of the public health defense strategy lay in geographical social distancing [2]. However, the unique characteristics of social media, such as diversification of information, liberalization of expression, and efficient transmission speed [3], had facilitated the propagation of a large number of rumors related to the spreading and blocking of the COVID-19 epidemic (eg, “The Air Force of the Central Theater District spreads disinfectant powder over Wuhan,” “Smoking and drinking can kill the new coronavirus,” and “From March 16th, citizen travel will be normalized”), resulting in public pressure and panic [4,5]. The probability of the public adopting constructive behavior (eg, maintaining personal hand hygiene and avoiding group aggregation) or disruptive behavior (eg, panic buying and adopting unproven treatments) largely depended on whether managers conveyed necessary deterministic information in a timely manner through online means to clarify rumors [6]. Evaluating the public’s reaction or attitude toward rumor rebuttal could help confirm the effectiveness of rumor rebuttal so as to guide the public to implement health decisions and actions based on the correct information [6,7].

While social media serves as a breeding ground for rumors, it is also directly used for rumor management [8-10]. However, the lack of fact verification of rumor rebuttal released by the public, as well as the national media or government organizations fabricating incorrect stories to conceal facts, lead to more intense controversy about rumor rebuttal, greatly dispelling the effectiveness of rumor rebuttal during public events [11]. In addition, the emergence of social media as an information dissemination channel shortens the distance from content producers to consumers and profoundly changes the way users obtain information, debate, and shape their attitudes [12]. Firstly, the information filtering mechanism based on algorithm recommendation technology mediates and promotes content promotion by considering users’ preferences and attitudes. Secondly, affected by individual and social factors, such as selective psychological mechanisms, group pressures, and social network circles, online users tend to choose the information that conforms to their belief system and ignore information that does not conform to their beliefs, eventually forming echo chambers, reflected as homophily-based communities of like-minded people that strengthen their shared narrative [13,14]. High segregation and clustering within this homophily-based community may increase the polarization of attitudes toward issues or events of public concern [15]. In the case of a user embedded in a community in which most users disagree with the rumor rebuttal, he or she is very likely to obey group norms, ignore the few voices within community that advocate refuting rumors, and even cut off communication with other communities holding rumor rebuttal views. Then, the scale of anti–rumor rebuttal groups continues to grow, and opposition groups are highly isolated. As a result, no matter how many repeated, mild attacks of rumor rebuttal are released, users’ ordinary beliefs (ie, agree or disagree with rumor rebuttal) are difficult to change [16,17]. Therefore, it is critical to explore how rumor rebuttal diffuses in a contemporary media environment where users can easily filter and choose their information sources.

### Goals of This Study

First, this research aimed to analyze the distribution of users’ attitudes (ie, agree, disagree, query, or unknown) toward rumor rebuttal related to COVID-19 on Chinese social media (ie, Weibo, the Chinese equivalent of Twitter [15]) to check its effectiveness. Second, we focused on determining whether and to what extent (ie, polarization based on users’ attitudes or even based on communities’ mixed attitudes) the echo chamber effect existed in the process of shaping users’ attitudes when retweeting or commenting on rumor rebuttal. Last but not least, we needed to evaluate the impact that the echo chamber effect had on the characteristics of interactive content. Specifically, using a combination of manual content analysis, automatic text analysis, social network analysis, and statistical analysis, we paid attention to retweeting and commenting networks at both the user and community levels, quantified the homophily based on the attitude distribution of nodes in networks, and checked the user interaction content from three dimensions: sentiment expression, information seeking and sharing, and civility. Generally, by understanding the community structure of the dissemination network of rumor rebuttal and the interactive nature of users within and among communities, we could comprehensively reveal the function and impact of echo chambers on guiding the public’s cognition as well as their decision-making and interactive behaviors.

### Prior Work

#### Rumor Rebuttal Dissemination and Echo Chamber Effect

Rumor spread refers to information being widely spread during uncertain or dangerous situations to alleviate fear and anxiety [18,19], while rumor rebuttal refers to the use of information to effectively combat rumors [20-23]. Research studies on the factors that influence the effectiveness of rumor rebuttal focus on the characteristics of the source subjects [10,24-27], content [28,29], and dissemination channels [30,31]. To some extent, these research studies ignore the individual differences in information receivers’ responses to rumor rebuttal and the context of the communities in which communicators are embedded. Limited research studies have pointed out that individuals with different knowledge reserves, interests, and values have different perceived credibility toward rumor rebuttal [32,33], and social identity plays a vital role in the transmission cascade of rumor rebuttal [34]. Individuals develop social identity by establishing cognitive and emotional connections with social groups, organizations, or other social entities, and they have a sense of belonging to entities with common beliefs [35]. This social identity is accompanied by confirmation bias; namely, individuals tend to shape their own attitudes to be in line with their prior attitudes as well as according to the standards that they share with the people around them in order to enhance their identity [34]. Such communities of judgment provide the basis for whether individuals view the decisions of their in-group as legitimate [34,36], eventually facilitating the emergence of echo chambers; namely, a kind of situation or circumstance where they tend to share ideas, information, or opinions with the same values, while barely considering alternative opinions [13,16]. From the perspective of social networks, the existence of clusters is understood as the evidence of echo chambers, in which nodes tend to preferentially connect to nodes in clusters and form homogeneous communities [37]. Homophily refers to the principle that contact between similar people occurs at a higher probability than between dissimilar people [38].

Based on users’ different interaction behaviors in different situations, different conclusions about the existence or exposure degree of the echo chamber effect are obtained. By analyzing political rumor rebuttal on Twitter during the 2012 US presidential election campaign, Shin et al [39] found that, within retweeting networks, rumor refuters neither formed a sizable community nor exhibited a partisan structure; that is, rejecters who supported rumor rebuttal about Barack Obama also engaged in debunking rumors about Mitt Romney—the two candidates were in competition with each other. In addition, Zollo et al [16] discovered the echo chamber effect in users’ interactions toward rumor rebuttal of unverified conspiracy information on Facebook: two well-formed and highly segregated communities existed around conspiracy and anticonspiracy topics (ie, users mainly liked or commented on only one category). Other research studies related to the echo chamber effect mainly focused on common and controversial public social issues, such as food safety [15], public advocacy [15,40], and climate change [41]. Less attention has been paid to the specific transmission process of rumor rebuttal, and even less research has been done to explore the public’s selective acceptance behavior of rumor rebuttal during public health emergencies. Relevant research conclusions need to be supplemented and verified.

Retweeting and commenting serve as evidence of the effectiveness of information dissemination strategies [10,42] or evidence of the significance of the content that was retweeted or commented on [43]. What’s more, retweets and comments can reflect public attitudes [15,40,41]. In addition, the nature of the online community may vary depending on the topic being discussed [38,41]. Based on the above trends and findings, we proposed the following research questions. Research Question 1 asks, “What was the distribution of attitudes (ie, agree, disagree, query, or unknown) toward rumor rebuttal under different topics related to COVID-19, and how effective was the rumor rebuttal?” Research Question 2 asks, “Based on the interactive mechanism of retweeting and commenting on Weibo, did the echo chamber effect exist in attitudes toward rumor rebuttal under different topics related to COVID-19? If so, to what extent?”

#### Sentiment, Information, Civility, and the Echo Chamber Effect

Informal networks on social media are used for different purposes, including sentiment expression, information seeking and sharing, and collective sensemaking [43]. Previous research studies on public sentiment during major public health emergencies found that people usually exhibited negative sentiments, such as panic, anxiety, anger, sadness, and disgust [44,45]. After rumors were refuted, public sentiment usually changed from negative to positive [46,47]. However, Zollo et al [16] found that negative sentiments dominated in the comments on Facebook that refuted conspiracy theories, regardless of users’ polarization. In addition, social identity theory proposed that identification with the entity involves positive feelings of sympathy about the entity and, conversely, that disidentification entails negative feelings of dislike or even hate [48]. In other words, the longer the discussions between polarized communities with opposite attitudes, the more negativity was found overall [49,50].

In addition to sharing personal experiences, cracking jokes, or expressing concern, people also use social media to ask questions or find and share knowledge [51]. On the one hand, affected populations actively seek explanations to reduce uncertainty in times of crisis [52]. On the other hand, the research on Twitter messages has found that over half of the posts were information related and contained links to websites [51,53].

Rowe [54] pointed out that the anonymity of the digital environment reduced the quality of online communication. When users participated in highly controversial debates, uncivil, discriminatory, hateful, and other remarks were more likely to surface [55]. Chen et al [56] argued that members with different attitudes might frequently retweet each other’s tweets, but meaningful or rational conversations seldom occur. In order to study the relationship between different types of user interactions and the sentiment-, information-, and civility-based tweets, we established the following research question. Research Question 3 asks, “Were the tweets from different kinds of interactions among users different in the distribution of sentiment tendency, information seeking or sharing, and civility?”

Berger [57] claimed that users experiencing amusement were more willing to share information. Wolleb et al [49] found that angry people who tended to exercise less critical judgment and relied more on stereotypes were more likely to seek out information confirming their prior attitudes to contribute to the echo chamber. Moreover, anger was also associated with incivility and hostility, which might increase distrust and polarization [58]. To study the relationship among sentiment, information, and civility, we put forward the following research question. Research Question 4 asks, “Were there any correlations when users expressed sentiment or sought or shared information when making uncivil remarks in rumor rebuttal discussions on Weibo?”

## Methods

**Figure 1:**
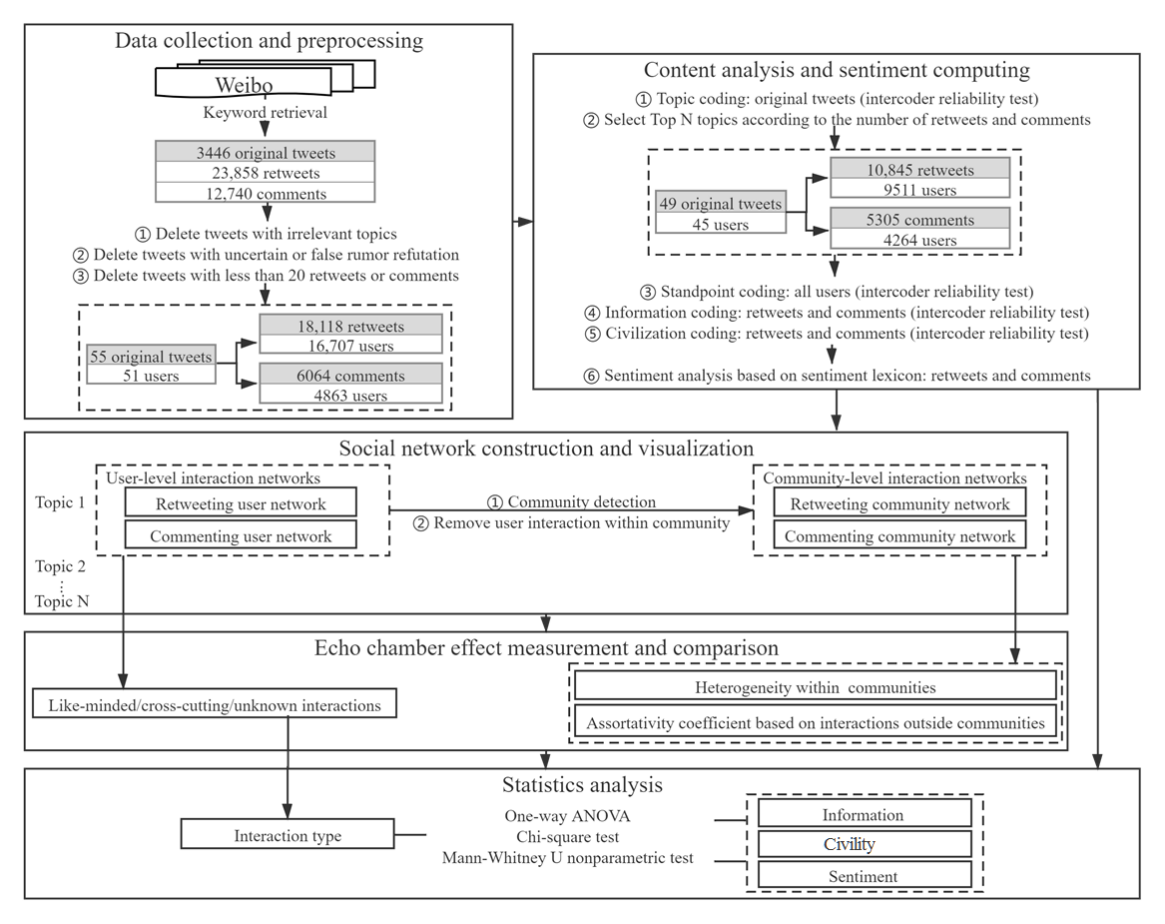
Research design. ANOVA: analysis of variance.

The research design for this study is outlined in Figure 1.

### Data Collection and Preprocessing

As human-to-human transmission has been confirmed [59], to prevent further spread of COVID-19 from its source, the city’s entire transportation system was prohibited from entering and leaving Wuhan starting from 10 AM on January 23, 2020, followed by the whole of Hubei province a day later. It was not until midnight on April 8, 2020, that Wuhan was unsealed to transportation. This period was the early stage of the COVID-19 epidemic. The high risk and uncertainty of the emerging infectious disease were likely to cause widespread public concern. Its suddenness and the insufficiency of the official response inevitably caused an information vacuum [60], which provided a breeding ground for rumors [46,61]. Due to geographical social distancing, rumor resolution mainly relied on online rebuttal. Therefore, this study first used Sina Weibo’s application programming interface [10] to crawl the original tweets containing the keywords “novel coronavirus (新冠)/COVID-19” and “rumor rebuttal (辟谣)” from 10 AM on January 23, 2020, to midnight on April 8, 2020; the posters’ information was also included. Next, we examined the list of retweets and comments of each original tweet to obtain the retweets and comments as well as the corresponding user information. We initially obtained 3446 original tweets, 23,858 retweets, and 12,740 comments on tweets.

Next, we performed data preprocessing. We deleted original tweets whose number of retweets or comments were below 20 to exclude low-impact samples [15]; we also deleted tweets that contained the above keywords but had nothing to do with the content in order to exclude noise from the data (eg, “#COVID-19#Rumor rebuttal is important”) [1]. Uncertain or false original rumor rebuttal tweets were also deleted. In addition to the excluded original tweets, we removed their retweets and comments. The final experimental data included 55 original rumor rebuttal tweets corresponding to 51 users, 18,118 retweets corresponding to 16,707 users, and 6064 comments corresponding to 4863 users.

### Content Analysis and Sentiment Computing

Firstly, we invited two trained professionals to create the topic categories for the 55 original rumor rebuttal tweets, referring to Chen’s topic classification rules for rumor rebuttal about the COVID-19 epidemic [30]. After adding and deleting certain topics, reviewing the topics, and eliminating disagreements during the actual coding process for the corpus, the topic categories were determined as shown in Table 1.

**Table 1:** Topic categories of original rumor rebuttal tweets.

Topic category	Explanation
Virus	The pathological characteristics of the virus, the name of the virus, etc
Contagion	The mode of disease transmission, the route of disease transmission, etc
Prevention	Disease prevention measures, related knowledge, etc
Patients	The physical health of the patients, the mental health of the patients, etc
Sequelae	The performance of sequelae in the recovered population
Epidemic situation	The spread of the epidemic in various regions
Domestic government countermeasures	Response measures of Chinese government departments at all levels
Other domestic countermeasures	Response measures of enterprises and organizations in China apart from the government
Foreign countermeasures	Response measures of countries outside China
Other	Other unimportant topics that were not equivalent to the above topics

Secondly, based on the number of retweets and comments and the number of users participating in retweeting and commenting under each topic, we selected the top number of topics (N) with the highest attention [10]. To answer Research Questions 1 and 2, we divided the attitudes of all the original posters, the retweeting users, and the commenting users who participated in the discussion under these topics into four categories: (1) agree—users agreed with the rumor rebuttal, (2) disagree—users disagreed with the rumor rebuttal, (3) query—users queried the rumor rebuttal, and (4) unknown—users had no clear attitude [62]. Attitudes of all the original posters were marked as *agree*. For retweeting users, we first determined the user’s attitude as expressed in a single retweet; we then comprehensively considered all of his or her retweets and selected the attitude that was most frequently expressed as the user’s attitude (ie, *agree*, *disagree*, or *query*). In the case of attitudes with equal frequencies, the attitude expressed by the latest retweet prevailed. The user’s attitude was coded as *unknown* if none of their retweets expressed his or her attitude clearly. The two coders coded 10% of the sample data and conducted intercoder reliability tests [63]. After eliminating differences and reaching agreement through discussion, they marked the remaining samples. By the same rule, they categorized the retweeting and commenting users, and intercoder reliability was calculated (retweeting users: κ=0.889; commenting users: κ=0.961). The high number of users who retweeted or commented on and agreed with the rumor rebuttal post served as a sign of effective refutation [10,64].

To analyze the content-related characteristics of retweets and comments, we coded them based on the dimensions of information (ie, seeking, sharing, and no information) and civility (ie, civility and incivility). The tweets that were abusive, threatening, or prejudiced against others or that were harmful to national laws and unity were classified as uncivil. The two dimensions were independent of each other. Intercoder reliability tests based on 10% of the sample data obtained κ values of 0.965 and 0.967 for information and civility of retweets, respectively, and 0.958 and 0.947 for information and civility of comments, respectively. All of the values were acceptable, suggesting that the results of the classification were robust.

Lastly, we adopted the sentiment analysis method based on the dictionary used by An and Ou [65], scored the sentiment intensity of sentiment-related words—scores of 1, 3, 5, 7, and 9, where 1 represented the lowest intensity and 9 represented the highest intensity—and considered the modification of negative words and adverbs on sentiment-related words. See equations 1 and 2 for details.

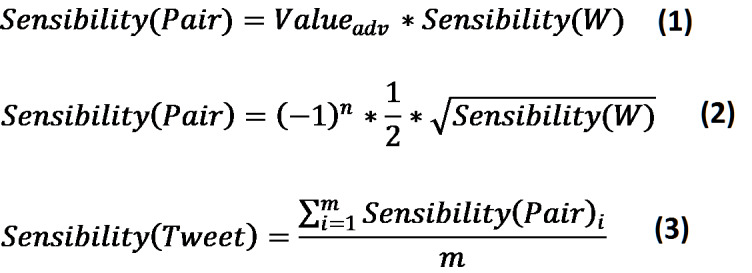



**Figure 2:**
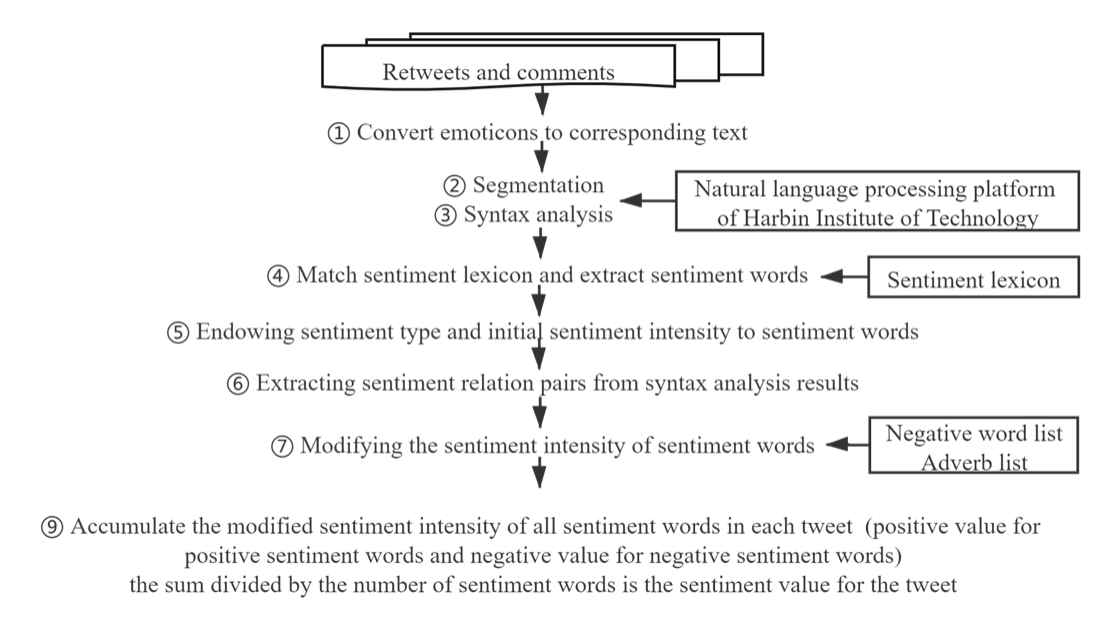
Sentiment computing.

*Sensibility(Pair)* was the modified sentiment intensity of the sentiment-related word. *Sensibility(W)* was the initial sentiment intensity of the sentiment-related word. *Value_adv_* was the intensity of the adverb (continuous value between 0 and 2) that modified the sentiment-related word. *n* was the number of negative words that modified the sentiment-related word. The sentiment scores of each retweet and comment were calculated (see equation 3; *m* was the number of sentiment-related words in each tweet). If the score was greater than 0, it was regarded as positive, and if the score was less than 0, it was regarded as negative; otherwise, it was neutral. Specific steps are shown in Figure 2.

### Social Network Construction and Visualization

#### User-Level Interaction Networks

To explore the structure of the user interaction network and its homophily at individual level, we established two user-level interaction networks based on retweeting and commenting behaviors under each topic (2 × N in total). In retweeting networks, if user *i* retweeted a tweet from user *j*, then there was an edge from *i* to *j*. In commenting networks, if user *i* commented on a tweet from user *j*, then there was an edge from *i* to *j*. Both retweeting and commenting networks were directed and weighted, and the weight of the edge was determined by the number of interactions between users. Networks were constructed using Python’s NetworkX package (Python Software Foundation) [66] to obtain the detailed features of topology structure and the homophily based on the users’ attitudes. Furthermore, we used Gephi’s Fruchterman Reinhold layout algorithm to visualize the connectivity and homophily of user networks [67].

#### Community-Level Interaction Networks

To explore the structure of users’ community networks and its homophily at the community level, in each user-level interaction network, we used Gephi’s community detection algorithm to divide each user into the corresponding community, where users interacted more frequently with each other than they did with others outside, based on the network topology and independent of the user’s attitude [68]. In 2 × N community-level interaction networks, each node represented a community and edges represented the remaining interactions between users who belonged to different communities after deleting the interactions within communities. Where *query* and *unknown* attitudes were equal to 0, *agree* was equal to 1, and *disagree* was equal to –1, we summarized and averaged the attitude scores of all users in each community, which represented the community’s attitude score [15,41].

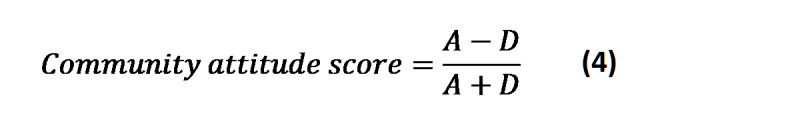



*A* was the observed frequency of members holding the a*gree* attitude and *D* was the observed frequency of members holding the *disagree* attitude within this community. The 2 × N directed weighted networks were constructed using Python’s NetworkX package [66] to obtain the detailed characteristics of topology structure and the homophily based on the community’s attitude score. Furthermore, we used Gephi’s Force Atlas layout algorithm to visualize the connectivity and homophily of community networks [67].

### Echo Chamber Effect Measurement

#### Echo Chamber Effect Based on User Level

Referring to Wang and Song [15], Tsai et al [40], and Williams et al [41], in each user-level interaction network, we counted the connection of nodes with the same or different attitudes and regarded the high connection frequency between similar nodes and/or low connection frequency between dissimilar nodes as evidence of homophily.

#### Echo Chamber Effect Based on Community Level

On the one hand, we analyzed heterogeneity within communities [15], which was defined as the balance between users holding *agree* and *disagree* attitudes within each community node and was measured as follows:

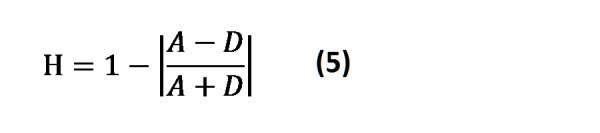



*A* was the observed frequency of members holding *agree* attitudes and *D* was the observed frequency of members holding *disagree* attitudes within this community. *H* gave values on a linear scale, ranging from perfect homogeneity (*H*=0, members holding only *agree* or only *disagree* attitudes) to perfect heterogeneity (*H*=1, equal proportions of members holding *agree* or *disagree* attitudes).

In addition, we also used Python’s NetworkX package [66] to calculate the assortativity coefficient *r* (–1 to 1) of each network based on the attitude scores of the community nodes and the interactions among communities. The coefficient measured the homophily of the network based on the node-level attribute (ie, the community’s attitude score), which essentially referred to the Pearson correlation of behaviors between the linked nodes [69-71]. If *r*>0, the node generally tended to connect to other nodes with similar properties and the network was called an assortative network; a larger *r* meant more prominent assortativity. If *r*≤0, this did not hold [15].

### Statistical Analysis

The Mann-Whitney *U* nonparametric test is mainly used to test whether there is a significant difference between the averages of two groups of samples [40,72]. One-way analysis of variance (ANOVA) is used to infer significant differences among three or more independent groups’ averages of a variable [15,73]. The chi-square test can be used for comparison of multiple rates or constituent ratios [74]. To answer Research Question 3, we used one-way ANOVA to compare the sentiment scores of retweets of like-minded, cross-cutting, unclear user interactions; we used the chi-square test to compare the proportion of retweets that contained information seeking, information sharing, and no information as well as civil and uncivil retweets of like-minded, cross-cutting, unclear user interactions. For commenting, we made the same comparison. To answer Research Question 4, we used one-way ANOVA to compare the sentiment scores of retweets that contained information seeking, information sharing, and no information; we used the Mann-Whitney *U* nonparametric test to compare the sentiment scores of retweets that were civil and uncivil; and we used the chi-square test to compare the proportion of civil and uncivil retweets that contained information seeking, information sharing, and no information. For commenting, we made the same comparison.

## Results

### Descriptive Statistics

The topic coding results for 55 original tweets are shown in Table 2. Apart from *others*, the top six topics that were widely retweeted and commented on by users were *epidemic situation*, *foreign countermeasures*, *prevention*, *virus*, *patients*, and *domestic government countermeasures*. The following research took the data corresponding to these six topics as the research object, including 49 original tweets published by 45 users, 10,845 retweets published by 9511 users, and 5305 comments published by 4264 users.

**Figure 3:**
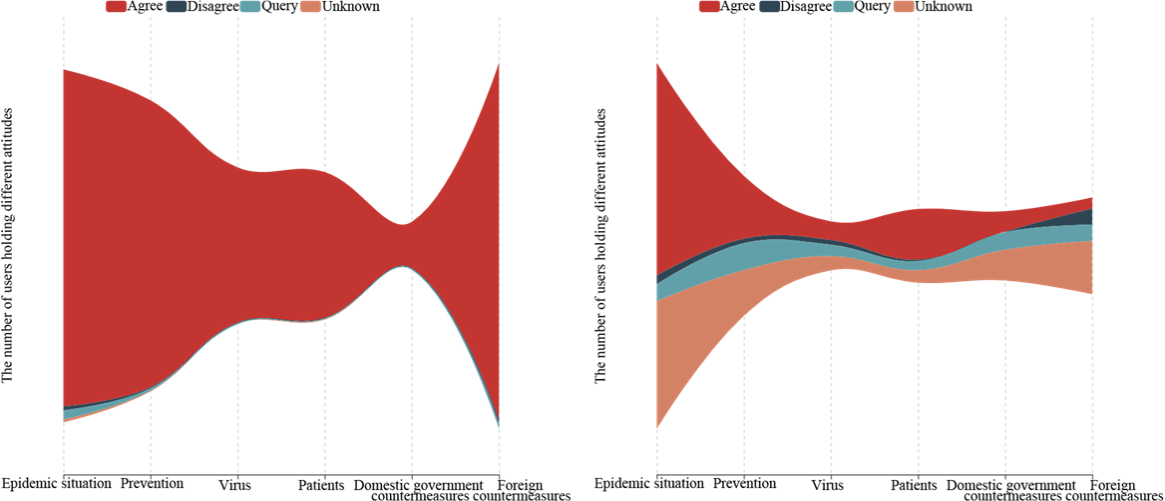
Distribution of attitudes of users who retweeted (left) or commented on (right) the original rumor rebuttal tweets under the top six topics.

In Figure 3, the different colors of the river branches represent different attitudes, and the widths of the river branches represent the number of users holding the corresponding attitudes. Most of the users who retweeted rumor rebuttal tweets displayed the *agree* attitude. Few users who retweeted rumor rebuttal tweets about the *epidemic situation* showed the *query* attitude. However, the users who commented on rumor rebuttal tweets had more diversified attitudes under various topics, with the majority of individuals holding *query* or *unknown* attitudes. Under the topics of *epidemic situation* and *foreign countermeasures*, especially, more users showed *disagree* or *query* attitudes.

**Table 2:** Topic distribution of original rumor rebuttal tweets and their retweets and comments.

Topic	Original tweets, n (%)^a^			Retweets, n (%)^a^			Comments, n (%)^a^			
	Users (n=51)	Tweets (n=55)	Users (n=16,707)		Retweets (n=18,118)	Users (n=4863)		Comments (n=6064)		
Virus	3 (6)	3 (5)	1204 (7.2)		1207 (6.7)	266 (5.5)		355 (5.8)		
Contagion	3 (6)	3 (5)	230 (1.4)		233 (1.3)	233 (4.8)		343 (5.7)		
Prevention	6 (12)	8 (15)	2235 (13.4)		2471 (13.6)	759 (15.6)		896 (14.8)		
Patients	4 (8)	4 (7)	1132 (6.8)		1137 (6.3)	399 (8.2)		414 (6.8)		
Treatment	0 (0)	0 (0)	0 (0)		0 (0)	0 (0)		0 (0)		
Sequelae	1 (2)	1 (2)	242 (1.4)		242 (1.3)	29 (0.6)		30 (0.5)		
Epidemic situation	25 (49)	25 (46)	2693 (16.1)		2835 (15.6)	1980 (40.7)		2603 (42.9)		
Domestic government countermeasures	5 (10)	5 (9)	364 (2.2)		372 (2.1)	374 (7.7)		462 (7.6)		
Other domestic countermeasures	1 (2)	1 (2)	25 (0.1)		25 (0.1)	30 (0.6)		35 (0.6)		
Foreign countermeasures	4 (8)	4 (7)	2813 (16.8)		2823 (15.6)	526 (10.8)		575 (9.5)		
Others	1 (2)	1 (2)	6773 (40.5)		6773 (37.4)	309 (6.4)		351 (5.8)		

^a^Percentages may add up to greater than 100% because one user may post multiple original tweets among which each tweet belongs to a specific topic.

### Echo Chamber Effect in Networks of Rumor Rebuttal Under Different Topics

#### Echo Chamber Effect in User-Level Interaction Networks

**Figure 4:**
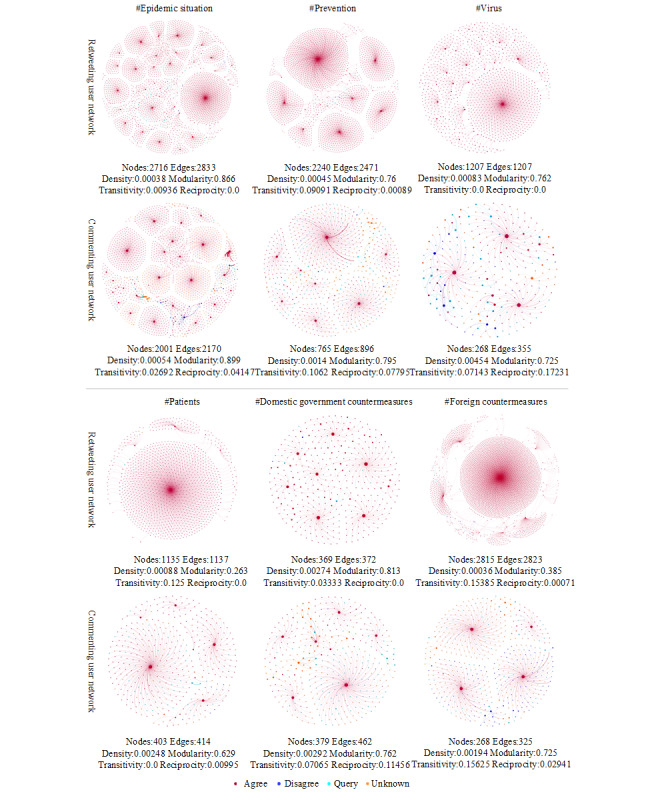
Retweeting user networks and commenting user networks of rumor rebuttal under the top six topics.

In Figure 4, retweeting user networks and commenting user networks are colored by users’ attitudes toward rumor rebuttal under the top six topics and visualized using Gephi’s Fruchterman Reingold layout algorithm. Each node’s size was proportional to its weighted in-degree, each line’s thickness was proportional to the edge’s weight, and each line’s color was consistent with the target node. Compared to retweeting networks, commenting networks were smaller but denser. Except for *virus* and *domestic government countermeasures*, the modularity values of commenting user networks under other topics were higher than those of retweeting user networks. Apart from *patients*, the transitivity and reciprocity—when the first individual chooses the second individual, the second individual also chooses the first individual—of commenting user networks under other topics were higher than those of retweeting user networks. Consequently, compared to retweeting users, commenting users created a more cohesive community with the help of the commenting mechanism [75], and the relationship between users was close and relatively stable [76].

**Figure 5:**
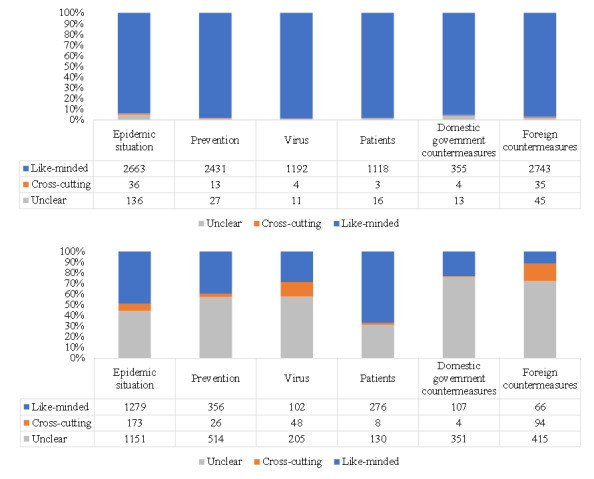
Homophily based on the users’ attitudes in retweeting and commenting user networks toward rumor rebuttal under the top six topics.

As shown in Figure 4, the retweeting and commenting user networks of rumor rebuttal under different topics showed highly modular structures; however, the large clusters in retweeting user networks showed high homophily, while the large clusters in commenting user networks had mixed attitudes. In addition, in retweeting user networks, the attitude of the user node located in the center was mostly *agree*, but in commenting user networks, it might be *agree*, *disagree*, or *query*. In Figure 5, *like-minded* refers to the sum of the edges’ weights in which the attitude of the nodes at both ends was either *agree* or *disagree*, *cross-cutting* refers to the sum of the edges’ weights in which the attitude of the nodes at one end was *agree* and at the other end was *disagree*, and *unclear* refers to the sum of the edges’ weights in which there was at least one node at both ends holding the *query* or *unknown* attitude. In retweeting user networks under different topics, the proportion of interactions between users with the same attitude ranged from 93.9% (2663/2835) to 98.8% (1192/1207), the proportion of interactions between users with opposite attitudes ranged from 0.3% (3/1137) to 1.3% (36/2835), and the proportion of interactions between users whose attitudes were not clear ranged from 0.9% (11/1207) to 4.8% (138/2835). In commenting user networks, the situation was quite different, with the like-minded interactions accounting for 11.5% (66/575) to 66.7% (276/414), cross-cutting interactions accounting for 0.9% (4/462) to 16.3% (94/575), and unclear interactions accounting for 31.4% (130/414) to 76.0% (351/462). These quantitative indicators revealed the significance of ideology echo chambers in retweeting user networks and the low homophily in commenting user networks.

#### Echo Chamber Effect in Community-Level Interaction Networks

**Figure 6:**
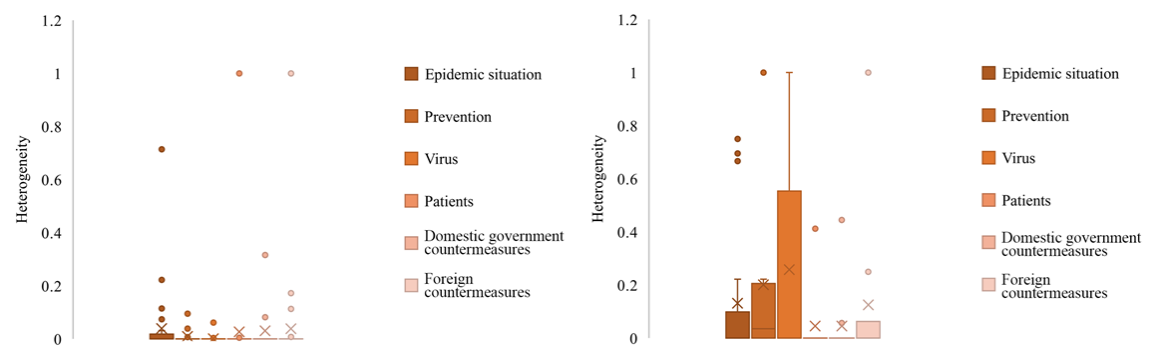
Heterogeneity based on the distribution of users' attitudes within each community node in retweeting (left) and commenting (right) community networks toward rumor rebuttal under the top six topics. The × symbols and circles represent means and outliers of the heterogeneity values, respectively.

Figure 6 indicates that the intracommunity heterogeneity value of the retweeting network under each topic was low. Except for *patients* and *domestic government countermeasures*, the intracommunity heterogeneity values of commenting networks under other topics were high. In addition, the intracommunity heterogeneity values of retweeting networks were generally lower than those of commenting networks.

**Figure 7:**
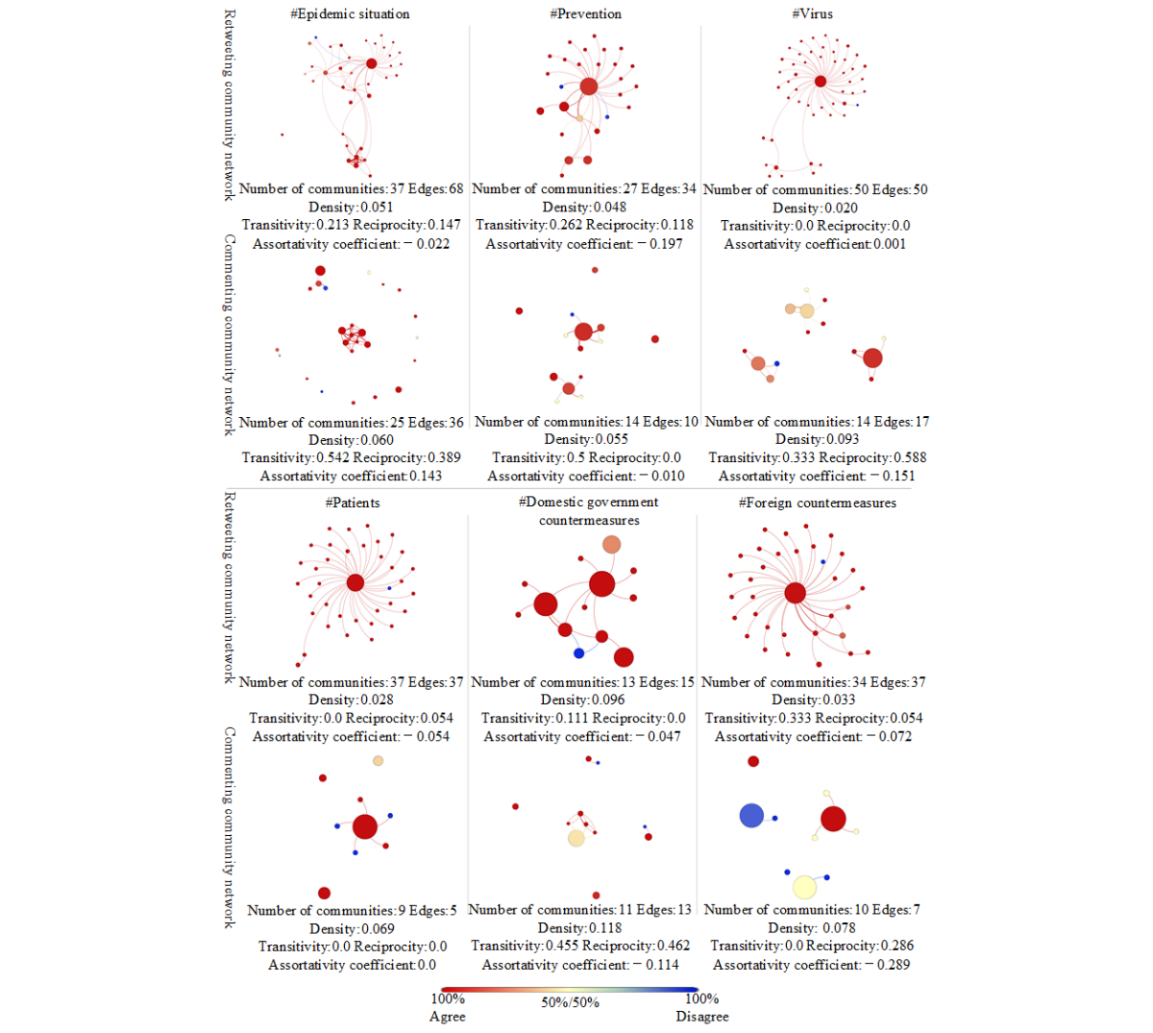
Retweeting community networks and commenting community networks.

In Figure 7, the retweeting and commenting community networks are colored by communities’ attitude scores toward rumor rebuttal under the top six topics; a score of 1 meant that 100% of users in the community held the *agree* attitude and a score of –1 meant that 100% of users in the community held the *disagree* attitude. These were visualized using Gephi’s Force Atlas layout algorithm. Each node’s size was proportional to the number of users within the community, each line’s thickness was proportional to the edge’s weight, and each line’s color was consistent with the target node. Compared to retweeting networks, the commenting community networks were smaller but denser and had larger nodes. Apart from *patients*, commenting community networks had higher transitivity; except for *patients* and *prevention*, they had higher reciprocity. This meant that, compared to retweeting networks, the interactions between communities in commenting networks were more common and the connections between communities were closer and more stable.

It was visually apparent that most communities in retweeting networks had strongly homogeneous attitude distributions toward rumor rebuttal, dominated by the *agree* attitude, while very few communities had a mix of opposite views. However, commenting networks were less segregated and showed more frequent occurrences of communities containing both *agree* and *disagree* attitudes. The assortativity coefficients of retweeting community networks ranged from –0.197 to 0.001, among which *virus* was the highest. The assortativity coefficients of commenting community networks ranged from –0.289 to 0.143, among which *epidemic situation* was the highest. The assortativity coefficients of the retweeting community networks under the topics of *virus*, *domestic government countermeasures*, and *foreign countermeasures* were higher than those of the commenting community networks. The assortativity coefficients of the commenting community networks under the topics of *epidemic situation*, *prevention*, and *patients* were higher than those of the retweeting community networks. All in all, on the one hand, the attitude distribution within communities of retweeting networks under all the topics and commenting networks under *patients* and *domestic government countermeasures* was homogeneous. On the other hand, the interaction homophily between communities in retweeting and commenting networks was low.

### The Impact of the Echo Chamber Effect on Information Characteristics of User Interaction Content

#### The Difference in Information Characteristics Between Interaction Types

**Figure 8:**
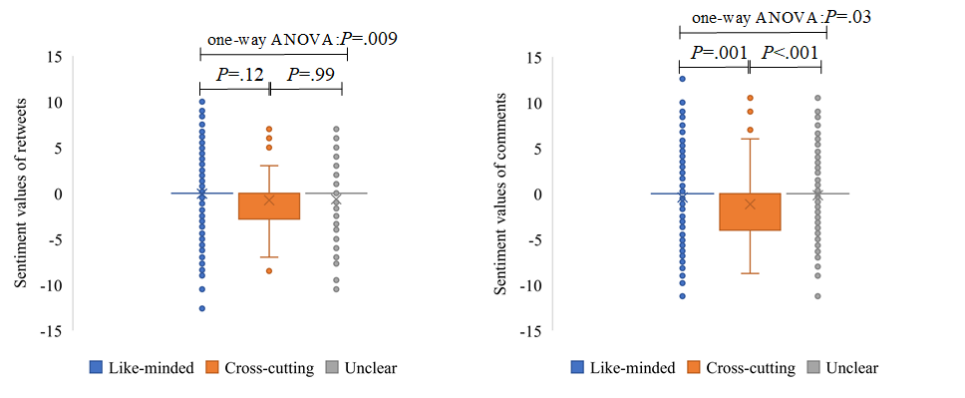
The distribution of sentiment values across different kinds of interactions in retweets (left) and comments (right). The × symbols and circles represent means and outliers of the sentiment values, respectively. ANOVA: analysis of variance.

As shown in Figure 8, the retweets and comments were generally negative, and the negative values in comments were higher. The average of the sentiment values in cross-cutting interactions was lower than that of the other two kinds of interactions. In addition, it is noteworthy that cross-cutting interactions of retweets and comments contained significantly higher negative values than did like-minded interactions.

**Figure 9:**
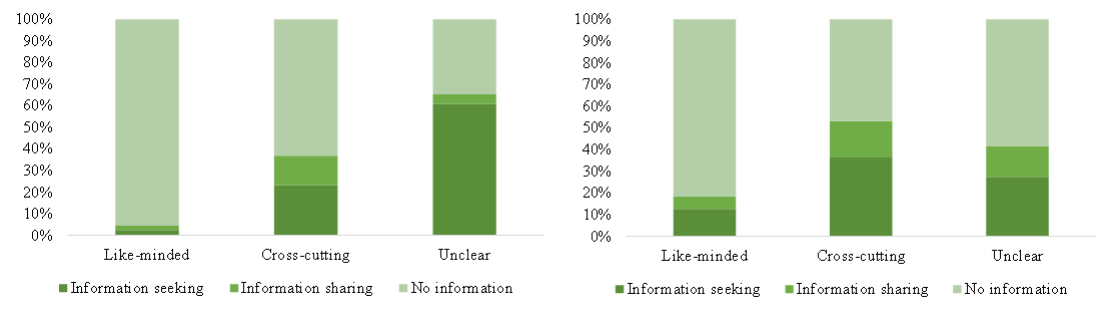
The proportion of information-seeking, information-sharing, and no information content in different kinds of interactions in retweets (left) and comments (right).

**Figure 10:**
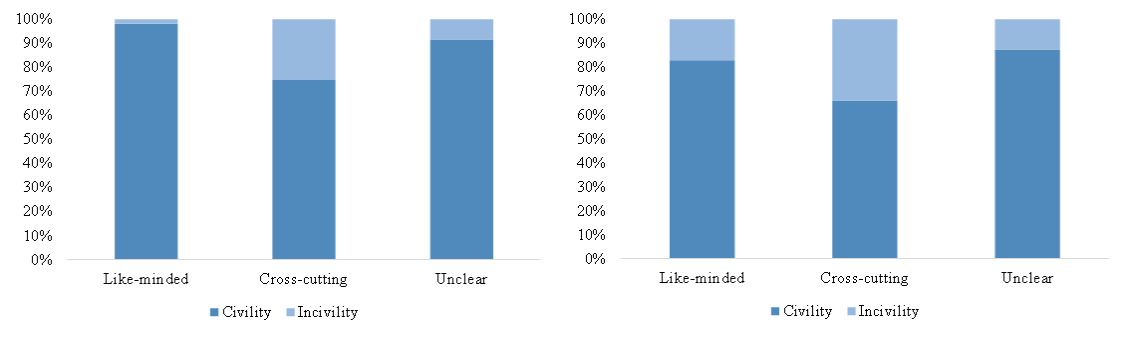
The proportion of civility and incivility in different kinds of interactions in retweets (left) and comments (right).

Figures 9 and 10 clearly state that about 4.0% (429/10,845) of the retweets contained information-seeking content and 2.1% (231/10,845) contained information-sharing content, while about 21.9% (1162/5305) of the comments contained information-seeking content and 10.9% (579/5305) contained information-sharing content. Only 2.2% (242/10,845) of the retweets contained uncivil expressions, while 15.9% (845/5305) of the comments contained uncivil expressions. The results of chi-square tests for both retweets and comments (*P*<.001) claimed that the proportion of information seeking, information sharing, and incivility in cross-cutting interactions was significantly higher than in like-minded interactions, at the significance level of .001.

#### Correlation of Different Information Characteristics

**Figure 11:**
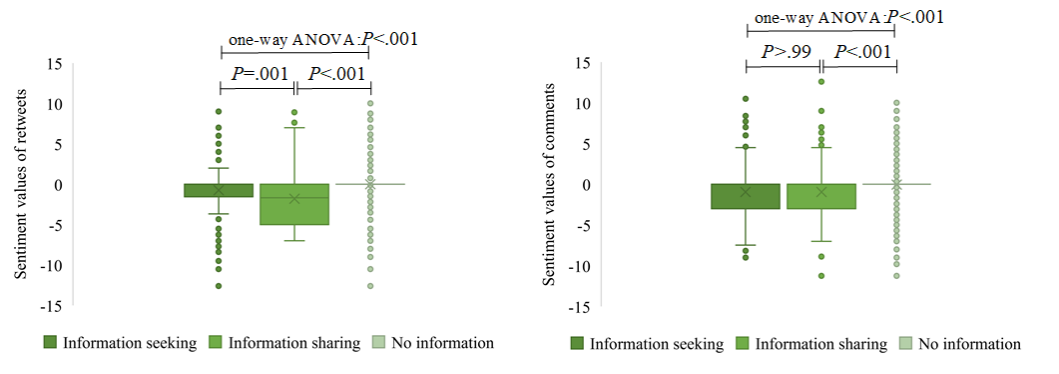
The distribution of sentiment values in retweets (left) and comments (right) containing information-seeking, information-sharing, and no information content. The × symbols and circles represent means and outliers of the sentiment values, respectively. ANOVA: analysis of variance.

**Figure 12:**
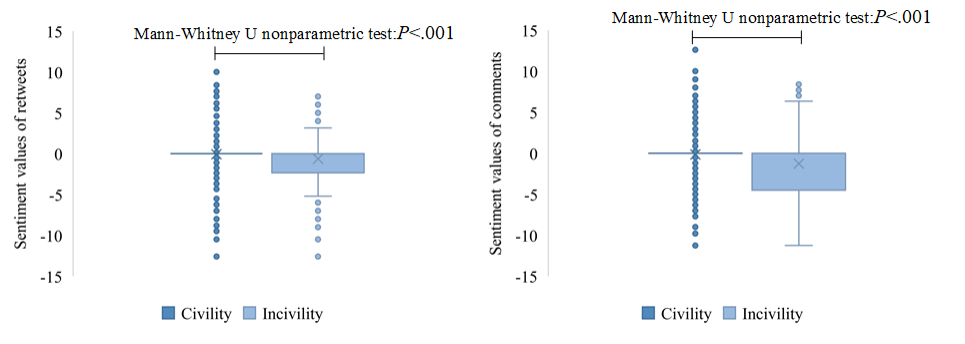
The distribution of sentiment values for retweets (left) and comments (right) containing civil and uncivil content. The × symbols and circles represent means and outliers of the sentiment values, respectively.

Figure 11 shows that the sentiment values of retweets or comments containing information-seeking and information-sharing content were significantly different. The average of the sentiment values of retweets without information-seeking and information-sharing content was the highest, and the average sentiment value containing information-sharing content was significantly lower than the one containing information-seeking content. A similar situation occurred with comments. Figure 12 shows that uncivil retweets or comments had higher negative sentiment values than civil retweets or comments.

**Figure 13:**
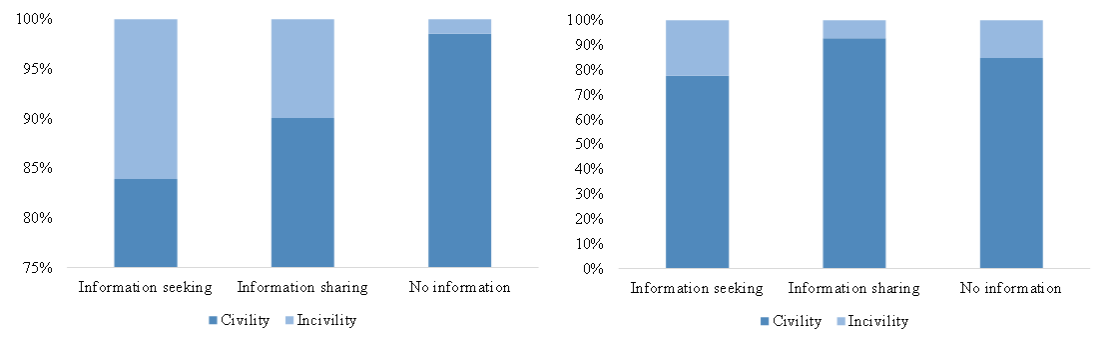
The proportion of civility and incivility in retweets (left) and comments (right) containing information-seeking, information-sharing, and no information content.

Figure 13 indicates that the proportion of incivility in tweets containing information-seeking content was highest, whether it was in retweets or comments. The results of chi-square tests (*P*<.001) illustrated that there were significant differences in the proportion of civility and incivility in comments containing information-seeking and information-sharing content, but there were no significant differences in retweets.

## Discussion

### Principal Findings

Considering the important role of the dissemination of rumor rebuttal on social media regarding rumor control and disease containment during public health crises, in this research, we used content analysis, sentiment analysis, social network analysis, and statistical analysis to roughly evaluate the effectiveness of rumor rebuttal, analyze the echo chamber effect based on attitudes in users’ retweeting and commenting behaviors toward rumor rebuttal under different topics, and analyze the impact of the echo chamber effect on information characteristics of user interaction content. Firstly, we analyzed the distribution of attitudes and whether there was an echo chamber effect in the attitude choice of individuals when retweeting or commenting on others’ tweets. Secondly, we tested the heterogeneity of attitude distribution within communities and the homophily of interactions between communities. Based on the results at user and community levels, we made a comprehensive judgment. Finally, we examined the content of user interaction from three dimensions of sentiment expression, information seeking and sharing, and civility to test the impact of the echo chamber effect.

### The Echo Chamber Effect in Rumor Rebuttal Communication

Retweeting indicates a desire to increase the visibility of a given message; comments are a way of online collective debate around the topic of a tweet [77]. Therefore, comments are more likely to include diversified pros and cons–related feedback on rumor rebuttal under different topics [16]. Moreover, social media users who participate in different topics of rumor rebuttal experience different degrees of the echo chamber effect in attitude selection based on different interaction mechanisms. The user-level interaction networks indicated a high frequency of like-minded interactions and a low frequency of cross-cutting interactions in retweeting networks of all topics; however, they indicated a low frequency of like-minded interactions and a high frequency of interactions containing users without clear attitudes in commenting networks of all topics. Consistent with the research of Wang and Song [15], Tsai et al [40], and Williams et al [41], our results once again emphasized the outstanding performance of attitude-based echo chambers in retweeting networks and the frequent interaction of dissimilar opinions in commenting networks. Retweeting often implies endorsement of either the individual tweet or its original author [41]. Thus, users tend to retweet others who have attitudes consistent with their own, which is in line with the goal of an individual developing social identity, while commenting usually serves as an open channel for viewpoint collision and fusion. However, our results might conflict with the research of Shin et al [39] and Zollo et al [16]. Results from the former study might be due to the influence of political context on selective exposure. Results from the latter study might lie in platform differences. As Facebook focuses on reciprocal social interaction and Weibo, like Twitter, concentrates more on the sharing of opinions with the reduction of social pressure brought about by anonymity, echo chambers exist in users’ commenting networks on Facebook and not on Weibo [78].

The community-level interaction networks strongly suggested that homophily occurred in attitude distribution within communities in retweeting networks under all topics and in commenting networks under *patients* and *domestic government countermeasures* topics. Like the conclusions from Williams et al [41] on the controversial topics about climate change, most individuals who engaged in online discussions were embedded within communities of like-minded users; such self-reinforcing *echo chambers* could prevent engagement with alternative attitudes and promote extreme views [79]. We also identified mixed-attitude communities in commenting networks corresponding to certain topics, in which users were frequently exposed to a diversity of attitudes. Williams et al [41] characterized such communities as *open forums*. Nevertheless, there was not a clear tendency for a whole community to interact most strongly with other communities of similar attitude composition either in retweeting or commenting networks. This indicated that the echo chamber effect was not significant at the level of community interaction.

### The Impact of the Echo Chamber Effect on Interaction Content

Online content of user interactions using different mechanisms had different properties. This reflected different motivations for using social media. Compared with retweeting, commenting was used more for sentiment expression, information seeking, and information sharing; it also contained more uncivil terms. These might be attributed to the comment mechanism acting as an *open forum* to reduce the individual’s exposure ratio in social networks [80]. Online social networks are recognized sites of both the construction of social identities [81,82] and their linguistic performance [83]. Social identity theory [81] asserts that willingness to negatively engage with out-group members is a way of affirming membership of the in-group. In other words, interactions with alternative attitudes are often accompanied by greater negative sentiment. In addition, to defend collective sensemaking, it is inevitable to breed uncivil terms. It is worth noting that most like-minded interactions did not seek or share information; on the contrary, cross-cutting interactions did seek or share information. This suggested that the echo chamber effect dealt with the potential adverse effects of knowledge flow and group wisdom gathering [15]. The research also showed that online users’ information-seeking behavior was accompanied by incivility, and information-sharing behavior was accompanied by a more negative sentiment, which was often accompanied by incivility. This was very detrimental to any kind of meaningful interaction between users.

### Theoretical Contributions and Practical Implications

There are three main contributions. Firstly, previous research studies on the effectiveness of rumor rebuttal were mostly based on self-reported perception and attitude data, and they ignored or simplified the actual network structure of rumor rebuttal dissemination and users’ cognition as well as their decision-making and interaction behaviors. This research analyzed the echo chamber effect in users’ responses to rumor rebuttal under different topics, based on naturally occurring online behavior data. This attempt filled the gap between rumor rebuttal and echo chamber research and provided a new research area. Secondly, when analyzing the echo chamber effect, this study started from multiple dimensions, such as topic, interaction mechanism, and interaction level, and used visualization combined with qualitative and quantitative indicators to enrich and enhance the robustness of the conclusions. Thirdly, this study not only determined the existence and degree of the echo chamber effect but also analyzed its impact on the characteristics of interactive content (ie, sentiment, information, and civility), introducing sentiment intensity to improve the accuracy of the analysis.

In the process of rumor rebuttal dissemination under different topics, the distribution of attitudes by users who retweeted or commented was different, and the significance of an attitude-based echo chamber effect was also different. Social media platform managers should systematically monitor users’ attitudes and should achieve multidimensional governance by topic, network (ie, retweeting and commenting), and community (ie, within the network community and between the network communities) in order to prevent or alleviate group polarization. Notably, managers should strengthen the sentiment guidance for users who denied the rumor rebuttal in the open commenting forum to guard against sentiment infection. Simultaneously, the censorship of uncivil tweets should be increased to update the network environment, so that information seeking and sharing can become more efficient.

### Limitations

It should be noted that Weibo’s @ function is often used to initiate conversations with target users. This kind of targeted request, different from nontargeted exposure of retweeting and commenting, may exhibit different degrees of the echo chamber effect and then affect the characteristics of interactive content. Additional research should be conducted in the future. This research showed that users’ sentiments were negative in retweets and comments. It is meaningful to explore how the echo chamber effect affects different types of negative sentiments, such as anger and fear.

### Conclusions

This study first analyzed the distribution of attitudes by users who retweeted or commented on rumor rebuttal on Weibo in the early stage of the COVID-19 epidemic and found that the effectiveness of rumor rebuttal varied among different topics. The study then dug deeply into the existence and degree of an attitude-based echo chamber effect and its impact on causing sentiment resonance, motivating information seeking and sharing, and breeding uncivil speech. The findings confirmed that retweeting played an essential role in promoting polarization, and commenting played a role in consensus building. The findings showed that there might not be a significant echo chamber effect in community interactions and verified that the echo chamber effect did have an essential impact on interactive content (ie, sentiment, information, and civility). Specifically, polarization caused cross-cutting interactions to contain more negative sentiment, which was associated with incivility, and caused like-minded interactions to contain less meaningful information seeking and sharing.

## Abbreviations

ANOVA: analysis of variance

## References

[1] Thelwall M, Thelwall S (2020). A thematic analysis of highly retweeted early COVID-19 tweets: Consensus, information, dissent and lockdown life. Aslib J Inf Manage.

[2] Tian H, Liu Y, Li Y, Wu C, Chen B, Kraemer M, Li B, Cai J, Xu B, Yang Q, Wang B, Yang P, Cui Y, Song Y, Zheng P, Wang Q, Bjornstad ON, Yang R, Grenfell BT, Pybus OG, Dye C (2020). An investigation of transmission control measures during the first 50 days of the COVID-19 epidemic in China. Science.

[3] Zubiaga A, Liakata M, Procter R, Wong Sak Hoi G, Tolmie P (2016). Analysing how people orient to and spread rumours in social media by looking at conversational threads. PLoS One.

[4] The Lancet (2020). COVID-19: Fighting panic with information. Lancet.

[5] Larson HJ (2020). Blocking information on COVID-19 can fuel the spread of misinformation. Nature.

[6] Lipsitch M, Swerdlow DL, Finelli L (2020). Defining the epidemiology of Covid-19 — Studies needed. N Engl J Med.

[7] Pal A, Banerjee S (2020). Internet users beware, you follow online health rumors (more than counter-rumors) irrespective of risk propensity and prior endorsement. Inf Technol People.

[8] Cao Y, An B (2011). Weibo gives rumors no time to thrive. China Daily.

[9] Zeng J, Chan CH, Fu KW (2017). How social media construct “truth” around crisis events: Weibo's rumor management strategies after the 2015 Tianjin blasts. Policy Internet.

[10] Zeng J, Burgess J, Bruns A (2019). Is citizen journalism better than professional journalism for fact-checking rumours in China? How Weibo users verified information following the 2015 Tianjin blasts. Glob Media China.

[11] Petrova P, Cialdini R (2005). Fluency of consumption imagery and the backfire effects of imagery appeals. J Consum Res.

[12] Del Vicario M, Bessi A, Zollo F, Petroni F, Scala A, Caldarelli G, Stanley HE, Quattrociocchi W (2016). The spreading of misinformation online. Proc Natl Acad Sci U S A.

[13] Dubois E, Blank G (2018). The echo chamber is overstated: The moderating effect of political interest and diverse media. Inf Commun Soc.

[14] Li W, Peng J (2019). Simulation experiment on echo chamber effect of social networks communication. Mod Commun.

[15] Wang X, Song Y (2020). Viral misinformation and echo chambers: The diffusion of rumors about genetically modified organisms on social media. Internet Res.

[16] Zollo F, Bessi A, Del Vicario M, Scala A, Caldarelli G, Shekhtman L, Havlin S, Quattrociocchi W (2017). Debunking in a world of tribes. PLoS One.

[17] Garrett RK (2017). The “echo chamber” distraction: Disinformation campaigns are the problem, not audience fragmentation. J Appl Res Mem Cogn.

[18] DiFonzo N, Bordia P (2007). Rumor Psychology: Social and Organizational Approaches.

[19] Jung C (1909). Contribution to the psychology of rumour. Indiana Assoc Health Phys Educ Recreat Dance J.

[20] Ozturk P, Li H, Sakamoto Y (2015). Combating rumor spread on social media: The effectiveness of refutation and warning. Proceedings of the 48th Hawaii International Conference on System Sciences.

[21] Tanaka Y, Sakamoto Y, Matsuka T (2013). Toward a social-technological system that inactivates false rumors through the critical thinking of crowds. Proceedings of the 46th Hawaii International Conference on System Sciences.

[22] Pal A, Chua AY, Goh DH (2017). Does KFC sell rat? Analysis of tweets in the wake of a rumor outbreak. Aslib J Inf Manage.

[23] Pal A, Chua AY, Hoe-Lian Goh D (2019). Debunking rumors on social media: The use of denials. Comput Human Behav.

[24] Berinsky AJ (2011). Rumors, truths, and reality: A study of political misinformation. Proceedings of the 69th Annual National Conference of the Midwest Political Science Association.

[25] Bordia P, DiFonzo N, Haines R, Chaseling E (2005). Rumors denials as persuasive messages: Effects of personal relevance, source, and message characteristics. J Appl Soc Psychol.

[26] Bordia P, DiFonzo N, Travers V (1998). Denying rumors of organizational change: A higher source is not always better. Commun Res Rep.

[27] DiFonzo N, Bordia P (2000). How top PR professionals handle hearsay: Corporate rumors, their effects, and strategies to manage them. Public Relat Rev.

[28] Chen J, Liu Y, Deng S (2017). Research on user reviews of government rumor-refuting information and factors influencing their emotional tendencies. Inf Sci China.

[29] Ruan W, Xia Z (2020). Analysis of influencing factors of social media users? Willingness to share counter-rumors. Sci Manage China.

[30] Chen Y (2020). Study on the spread and control of internet rumors of public health emergencies: Text analysis of internet rumors based on COVID-19 epidemic. E-Government.

[31] Huang J (2020). Rumor rebuttal practice of government WeChat during COVID-19 epidemic: A case study of "Ruian release". J News Res China.

[32] Li Z, Zhang Q, Wang Y, Wang S (2020). Social media rumor refuter feature analysis and crowd identification based on XGBoost and NLP. Appl Sci (Basel).

[33] Anthony S (1992). The influence of personal characteristics on rumor knowledge and transmission among the deaf. Am Ann Deaf.

[34] Einwiller S, Kamins M (2008). Rumor has it: The moderating effect of identification on rumor impact and the effectiveness of rumor refutation. J Appl Soc Psychol.

[35] Mael F, Ashforth BE (1992). Alumni and their alma mater: A partial test of the reformulated model of organizational identification. J Organ Behav.

[36] DiFonzo N, Uscinski JE (2018). Conspiracy rumor psychology. Conspiracy Theories and the People Who Believe Them.

[37] Bruns A (2017). Echo chamber? What echo chamber? Reviewing the evidence. Proceedings of the 6th Biennial Future of Journalism Conference (FOJ17).

[38] Himelboim I, McCreery S, Smith M (2013). Birds of a feather tweet together: Integrating network and content analyses to examine cross-ideology exposure on Twitter. J Comput Mediat Commun.

[39] Shin J, Jian L, Driscoll K, Bar F (2016). Political rumoring on Twitter during the 2012 US presidential election: Rumor diffusion and correction. New Media Soc.

[40] Tsai WS, Tao W, Chuan C, Hong C (2020). Echo chambers and social mediators in public advocacy issue networks. Public Relat Rev.

[41] Williams HTP, McMurray JR, Kurz T, Lambert FH (2015). Network analysis reveals open forums and echo chambers in social media discussions of climate change. Glob Environ Change.

[42] Wang B, Zhuang J (2017). Crisis information distribution on Twitter: A content analysis of tweets during Hurricane Sandy. Nat Hazards.

[43] David CC, Ong JC, Legara EFT (2016). Tweeting supertyphoon Haiyan: Evolving functions of Twitter during and after a disaster event. PLoS One.

[44] Person B, Sy F, Holton K, Govert B, Liang A, National Center for Inectious Diseases/SARS Community Outreach Team (2004). Fear and stigma: The epidemic within the SARS outbreak. Emerg Infect Dis.

[45] Yang JZ, Chu H (2016). Who is afraid of the Ebola outbreak? The influence of discrete emotions on risk perception. J Risk Res.

[46] Dong W, Tao J, Xia X, Ye L, Xu H, Jiang P, Liu Y (2020). Public emotions and rumors spread during the COVID-19 epidemic in China: Web-based correlation study. J Med Internet Res.

[47] Zeng R, Zhu D (2019). A model and simulation of the emotional contagion of netizens in the process of rumor refutation. Sci Rep.

[48] Einwiller SA (2006). Enough is enough! When identification no longer prevents negative corporate associations. J Acad Mark Sci.

[49] Wollebæk D, Karlsen R, Steen-Johnsen K, Enjolras B (2019). Anger, fear, and echo chambers: The emotional basis for online behavior. Soc Media Soc.

[50] Xu W, Zhang C (2018). Sentiment, richness, authority, and relevance model of information sharing during social crises—The case of #MH370 tweets. Comput Human Behav.

[51] Chew C, Eysenbach G (2010). Pandemics in the age of Twitter: Content analysis of tweets during the 2009 H1N1 outbreak. PLoS One.

[52] Merrifield N, Palenchar M (2012). Uncertainty reduction strategies via Twitter: The 2011 wildfire threat to Los Alamos National Laboratory. Proceedings of the Association for Education in Journalism and Mass Communication (AEJMC) Annual Conference.

[53] Hughes AL, Palen L (2009). Twitter adoption and use in mass convergence and emergency events. Int J Emerg Manag.

[54] Rowe I (2014). Civility 2.0: A comparative analysis of incivility in online political discussion. Inf Commun Soc.

[55] Berg J (2016). The impact of anonymity and issue controversiality on the quality of online discussion. J Inf Technol Polit.

[56] Chen L, Wu X, Li M (2018). Formation and fragmentation within a networked public sphere: Social media debates on Traditional Chinese Medicine. Telematics Inform.

[57] Berger J (2011). Arousal increases social transmission of information. Psychol Sci.

[58] Hasell A, Weeks BE (2016). Partisan provocation: The role of partisan news use and emotional responses in political information sharing in social media. Hum Commun Res.

[59] Wang C, Horby PW, Hayden FG, Gao GF (2020). A novel coronavirus outbreak of global health concern. Lancet.

[60] Yang J, Lee S (2020). Framing the MERS information crisis: An analysis on online news media's rumour coverage. J Conting Crisis Manage.

[61] Gu H, Chen B, Zhu H, Jiang T, Wang X, Chen L, Jiang Z, Zheng D, Jiang J (2014). Importance of internet surveillance in public health emergency control and prevention: Evidence from a digital epidemiologic study during avian influenza A H7N9 outbreaks. J Med Internet Res.

[62] Ma J, Luo Y (2019). The classification of rumour standpoints in online social network based on combinatorial classifiers. J Inf Sci.

[63] Krippendorff K (2011). Computing Krippendorff's alpha-reliability. University of Pennsylvania ScholarlyCommons.

[64] Li Z, Zhang Q, Du X, Ma Y, Wang S (2021). Social media rumor refutation effectiveness: Evaluation, modelling and enhancement. Inf Process Manag.

[65] An L, Ou M (2017). Social network sentiment map of the stakeholders in public health emergencies. Libr Inf Serv China.

[66] Hagberg AA, Schult DA, Swart PJ (2008). Exploring network structure, dynamics, and function using NetworkX. Proceedings of the 7th Python in Science Conference (SciPy2008).

[67] Grandjean M (2015). GEPHI – Introduction to network analysis and visualization. Serval, Université de Lausanne.

[68] Kauffman J, Kittas A, Bennett L, Tsoka S (2014). DyCoNet: A Gephi plugin for community detection in dynamic complex networks. PLoS One.

[69] Newman MEJ (2002). Assortative mixing in networks. Phys Rev Lett.

[70] Noldus R, Van Mieghem P (2015). Assortativity in complex networks. J Complex Netw.

[71] Thedchanamoorthy G, Piraveenan M, Kasthuriratna D, Senanayake U (2014). Node assortativity in complex networks: An alternative approach. Procedia Comput Sci.

[72] McKnight PE, Najab J, Weiner IB, Craighead WE (2010). Mann-Whitney U test. The Corsini Encyclopedia of Psychology. 4th edition.

[73] Bewick V, Cheek L, Ball J (2004). Statistics review 9: One-way analysis of variance. Crit Care.

[74] Berkson J (1938). Some difficulties of interpretation encountered in the application of the chi-square test. J Am Stat Assoc.

[75] Kartun-Giles AP, Bianconi G (2019). Beyond the clustering coefficient: A topological analysis of node neighbourhoods in complex networks. Chaos Solitons Fractals: X.

[76] Weng L, Menczer F, Ahn Y (2013). Virality prediction and community structure in social networks. Sci Rep.

[77] Ellison N, Steinfield C, Lampe C (2007). The benefits of Facebook "friends": Social capital and college students' use of online social network sites. J Comput Mediat Commun.

[78] Hughes DJ, Rowe M, Batey M, Lee A (2012). A tale of two sites: Twitter vs Facebook and the personality predictors of social media usage. Comput Human Behav.

[79] Sunstein CR (2007). Republic.com 2.0.

[80] Liu Y, Jin X, Shen H, Cheng X (2017). Do rumors diffuse differently from non-rumors? A systematically empirical analysis in Sina Weibo for rumor identification. Proceedings of the 21st Pacific-Asia Conference on Knowledge Discovery and Data Mining.

[81] Tajfel H, Turner J, Austin WG, Worchel S (1979). An integrative theory of intergroup conflict. The Social Psychology of Intergroup Relations.

[82] Zhao S, Grasmuck S, Martin J (2008). Identity construction on Facebook: Digital empowerment in anchored relationships. Comput Human Behav.

[83] Tamburrini N, Cinnirella M, Jansen VA, Bryden J (2015). Twitter users change word usage according to conversation-partner social identity. Soc Networks.

